# MyD88-dependent Toll-like receptor 2 signaling modulates macrophage activation on lysate-adsorbed Teflon™ AF surfaces in an *in vitro* biomaterial host response model

**DOI:** 10.3389/fimmu.2023.1232586

**Published:** 2023-08-24

**Authors:** Laura A. McKiel, Laurel L. Ballantyne, Gian Luca Negri, Kimberly A. Woodhouse, Lindsay E. Fitzpatrick

**Affiliations:** ^1^ Department of Chemical Engineering, Faculty of Engineering and Applied Sciences, Queen’s University, Kingston, ON, Canada; ^2^ Centre for Health Innovation, Queen’s University and Kingston Health Sciences, Kingston, ON, Canada; ^3^ Independent Researcher, Kingston, ON, Canada; ^4^ Department of Biomedical and Molecular Sciences, Faculty of Health Sciences, Queen’s University, Kingston, ON, Canada

**Keywords:** biomaterials, macrophage, toll-like receptors, damage-associated molecular patterns, protein adsorption, foreign body reaction, insulin infusion cannulas, polytetrafluoroethylene

## Abstract

The adsorbed protein layer on an implanted biomaterial surface is known to mediate downstream cell-material interactions that drive the host response. While the adsorption of plasma-derived proteins has been studied extensively, the adsorption of damage-associated molecular patterns (DAMPs) derived from damaged cells and matrix surrounding the implant remains poorly understood. Previously, our group developed a DAMP-adsorption model in which 3T3 fibroblast lysates were used as a complex source of cell-derived DAMPs and we demonstrated that biomaterials with adsorbed lysate potently activated RAW-Blue macrophages via Toll-like receptor 2 (TLR2). In the present study, we characterized the response of mouse bone marrow derived macrophages (BMDM) from wildtype (WT), TLR2^-/-^ and MyD88^-/-^ mice on Teflon™ AF surfaces pre-adsorbed with 10% plasma or lysate-spiked plasma (10% w/w total protein from 3T3 fibroblast lysate) for 24 hours. WT BMDM cultured on adsorbates derived from 10% lysate in plasma had significantly higher gene and protein expression of IL-1β, IL-6, TNF-α, IL-10, RANTES/CCL5 and CXCL1/KC, compared to 10% plasma-adsorbed surfaces. Furthermore, the upregulation of pro-inflammatory cytokine and chemokine expression in the 10% lysate in plasma condition was attenuated in TLR2^-/-^ and MyD88^-/-^ BMDM. Proteomic analysis of the adsorbed protein layers showed that even this relatively small addition of lysate-derived proteins within plasma (10% w/w) caused a significant change to the adsorbed protein profile. The 10% plasma condition had fibrinogen, albumin, apolipoproteins, complement, and fibronectin among the top 25 most abundant proteins. While proteins layers generated from 10% lysate in plasma retained fibrinogen and fibronectin among the top 25 proteins, there was a disproportionate increase in intracellular proteins, including histones, tubulins, actins, and vimentin. Furthermore, we identified 7 DAMPs or DAMP-related proteins enriched in the 10% plasma condition (fibrinogen, apolipoproteins), compared to 39 DAMPs enriched in the 10% lysate in plasma condition, including high mobility group box 1 and histones. Together, these findings indicate that DAMPs and other intracellular proteins readily adsorb to biomaterial surfaces in competition with plasma proteins, and that adsorbed DAMPs induce an inflammatory response in adherent macrophages that is mediated by the MyD88-dependent TLR2 signaling pathway.

## 1 Introduction

The immune response to biomaterial implants, known as the foreign body reaction (FBR), is a significant challenge in the biomedical engineering field (1). The FBR describes a chronic inflammatory response to an implanted material or device, which culminates in the fibrous encapsulation of the implant (1). For certain applications, including some drug delivery devices, the fibrotic capsule prevents the implant from performing its intended function, resulting in implant failure (1). The FBR is initiated upon material implantation and the associated tissue damage. The implant surface rapidly adsorbs proteins from the surrounding fluid phase that contains both blood released from damaged blood vessels and contents released from damaged cells in the implant microenvironment, which includes damage-associated molecular patterns (DAMPs) (1). Neutrophils and then macrophages are recruited to the implant site, and interact with the implant via this adsorbed protein layer (1). Macrophages are known to be key players in the progression of the FBR; they are present at the implant site as early as 24 hours and remain for the lifetime of the implant, they fuse to form foreign body giant cells (FBGC) that are hallmarks of the FBR, and they orchestrate further leukocyte recruitment and downstream tissue remodeling events through paracrine signaling (2).

Due to its impact on the performance and longevity of long-term implants, fibrous capsule formation is frequently the target of research that aims to understand molecular mechanisms that drive the FBR and develop strategies for reducing or eliminating this adverse host response. However, short-term implants, such as glucose sensors and insulin infusion sets (IIS) used in insulin pump therapy, are also adversely impacted by the host response long before fibrosis occurs. In 2019, the Centers for Disease Control and Prevention (CDC) estimated that there were 1.6 million people in the United States of America living with type 1 diabetes (T1D) (3) and, of these, approximately 30 to 40% use insulin pump technology (e.g. continuous subcutaneous insulin infusion (CSII) systems) to deliver insulin and manage blood glucose levels (4). However, there are many challenges with CSII, including complications related to the IIS (5). The IIS consists of a polytetrafluorethylene (PTFE; tradename Teflon™) or stainless-steel cannula that is inserted into the subcutaneous fat and delivers an insulin analogue solution from the pump to the subcutaneous tissue. Most IIS are approved to be worn for 2 - 3 days, while a newly approved extended infusion set (EIS) can be worn up to 7 days (5). Beyond the recommended wear time, and sometimes even within this period, insulin delivery can become inconsistent, rapidly leading to potentially dangerous side effects of unexplained hyperglycemia and diabetic ketoacidosis (5–7). Emerging evidence suggests that the challenges with variable insulin adsorption in CSII are due, in part, to the acute inflammatory response at the insulin infusion site (8–10).

The acute inflammatory response to biomaterials, including IIS, is characterized by the early events of protein adsorption and macrophage adhesion, activation, and fusion on the material surface. Adsorption of blood-derived proteins on biomaterials surfaces and the response of macrophages to blood-derived adsorbed protein layers has been extensively investigated since the early 1970’s, and have focused primarily on adsorption of a handful of plasma proteins, such as albumin, immunoglobulin-γ, fibrinogen, high molecular weight kininogen, complement C3, lipoproteins, fibronectin and vitronectin (11–21). The introduction of proteomic analysis of adsorbed protein layers on biomaterial surfaces has clearly demonstrated that adsorbed protein profiles are significantly more diverse than originally reported but continue to focus predominantly on *in vitro* protein adsorption models using plasma or serum (22–26). However, one proteomic study of proteins adsorbed to the polyethylene glycol (PEG)-based hydrogels *in vivo* demonstrated proteins from the intracellular compartment and extracellular matrix also adsorb within the protein layer (27). In this study, we focus instead on the *in vitro* adsorption of tissue damage products, collectively referred to as DAMPs, and the response of primary mouse bone marrow derived macrophages. This study builds upon previous work from our group demonstrating that DAMP-containing fibroblast lysates adsorb to polymeric surfaces in the presence of blood proteins and induce a pro-inflammatory and pro-fibrotic response in the RAW264.7 and RAW-Blue mouse macrophage cell lines over 120 hours, which mimics the cytokine secretion profile and macrophage fusion of *in vivo* macrophage-material interactions (28, 29). Furthermore, this pro-inflammatory response was shown to occur primarily through Toll-like receptor 2 (TLR2) signaling (28–30). TLR2 is a cell surface TLR that, upon ligation, forms a heterodimer with either TLR1 or TLR6 and induces the myeloid differentiation primary-response gene 88 (MyD88)-dependent activation of nuclear factor-кB (NF-кB) transcription factors, and the production of pro-inflammatory cytokines, such as tumour necrosis factor α (TNF-α), interleukin 1 beta (IL-1β), and interleukin 6 (IL-6) (31). Other research has also implicated the TLR adaptor protein MyD88 as a critical factor mediating fibrous capsule formation surrounding subcutaneous implants in mice (32). Therefore, we sought to investigate TLR2 and MyD88 as potential targets for reducing the severity of FBR and its impact on biomedical devices and implants.

While role of DAMPs and TLRs in the induction of sterile inflammatory responses is well established (33), the relative importance of TLR signaling in biomaterial host responses remains unclear. Therefore, in this work we evaluated macrophage cultured on model PTFE surfaces using an *in vitro* protein adsorption model that incorporates DAMPs within a plasma-derived protein layer (28) and investigated the role of TLR2- and MyD88-dependent signaling pathways. We first characterized the responses of primary bone marrow derived macrophages (BMDMs) from wildtype (WT), TLR2 knockout (TLR2^-/-^), and MyD88 knockout (MyD88^-/-^) mice to Teflon™ AF surfaces with adsorbed DAMPs and plasma proteins. We then characterized the profile of adsorbed proteins derived from plasma or lysate-spiked plasma using mass spectrometry (MS)-based proteomics to explore what lysate-derived proteins adsorbed within the plasma protein layer and identify potential DAMPs that may contribute to the activation of surface adherent macrophage via TLR2/MyD88 signaling. Plasma anticoagulated with calcium chelators (e.g. K2 EDTA, citrate) were used in this study to inactivate both the complement and coagulation cascades, thus enabling the effect of DAMPs within the adsorbed protein layer to be elucidated. These *in vitro* results provide evidence that TLR-dependent signaling contributes to the acute inflammatory response to model Teflon™ AF surfaces, and merits further investigation into its ability to modulate FBR.

## 2 Materials and methods

### 2.1 Teflon™ AF surface preparation

Amorphous fluoropolymer Teflon™ AF 1600 (Sigma-Aldrich, St. Louis, MO), hereafter referred to simply as Teflon™ AF, was used as a cell culture substrate to model the commercial PTFE IIS cannulas. Teflon™ AF is a copolymer of 65 mol% 2-bistrifluoromethyl-4,5-difluoro-1,3-dioxole (PDD) and 35 mol% tetrafluoroethylene (TFE), and has previously been used to model PTFE surfaces by our group (29, 30) and others (34, 35) due to its similar characteristics to PTFE, including wettability (36). However, while both Teflon™ AF and PTFE are fluoropolymers, they have different surface chemistries due to the oxygen content of the PDD comonomer in Teflon™ AF. Teflon™ AF can be easily incorporated into cell culture systems, as it is soluble in perfluorinated solvents and can be cast from solution (29, 30). Furthermore, the amorphous structure of Teflon™ AF imparts excellent optical clarity, which is beneficial when visualizing adherent cells.

Teflon™ AF was dissolved in a fluorinated solvent (FC-40, Sigma-Aldrich) at 1 mg/mL and coated onto 6 well polystyrene plates, using the protocol originally developed by the Grainger group (35). Plates were dried in a vacuum oven (50 cmHg, 40 °C) for 48 hours to remove solvent. The wells were then cleaned with 70% (by volume) ethanol for one hour, followed by 30 minutes of ultraviolet (UV) sterilization (30, 35). Endotoxin-free water washes were performed on the wells for 1 hour (three times), 12 hours, and 24 hours prior to use to remove any remaining solvent. All batches of Teflon™ AF-coated wells were tested indirectly for endotoxin (n = 3 per batch, plated in duplicate) with a LAL Pyrochrome kit (CapeCod and Associates, East Falmouth, MA), and endotoxin levels were consistently below 0.05 EU/mL. Details on the indirect endotoxin assay methods have been previously described (30).

### 2.2 Plasma and lysate preparations

Innovative Grade US Origin Mouse C57BL6 Plasma (InnovativeResearch, Novi, MI) was used to generated adsorbed protein layers (10% plasma and 10% lysate in plasma) for macrophage experiments. For the proteomic analysis, citrated mouse plasma from C57BL/6J mice, generously provided by Prof. David Lillicrap (Queen’s University, Kingston, ON, Canada), was used. Mouse fibroblast lysate was generated by freeze-thaw cycling mouse NIH3T3 fibroblasts (ATCC, Manassas, VA), as described previously (28, 30). Briefly, NIH3T3 murine fibroblasts were maintained in Dulbecco’s modified Eagle’s medium (DMEM; Sigma-Aldrich, St. Louis, MO) containing 10% fetal bovine serum (FBS; Wisent, St. Bruno, QC) and 1% penicillin/streptomycin. To generate lysate, fibroblasts were washed in phosphate buffered saline (PBS; Gibco, Waltham, MA), resuspended at 5 x10^6^ cells/mL in PBS, and freeze−thaw cycled three times in a −80°C freezer and 37°C water bath. The total protein concentration of plasma and lysate was quantified using a microBCA assay (Thermo Scientific, Waltham, MA) according to manufacturer instructions, and protein solutions were aliquoted and stored at −80°C for future use.

### 2.3 Protein adsorption on Teflon™ AF surfaces

Mouse plasma was diluted to 10 vol% in PBS and was referred to hereafter as “10% plasma” or abbreviated as “Pla”. Lysate was spiked into the 10% plasma solution, such that lysate made up 10% of the total protein concentration and was referred to hereafter as “10% lysate in plasma” or abbreviated as “LysPla”. Teflon™ AF-coated 6 well plates were pre-conditioned with 10% plasma (420 μg/cm^2^), 10% lysate in plasma (420 μg total protein/cm^2 = ^42 μg lysate protein/cm^2 + ^378 μg plasma protein/cm^2^), or assay media (RPMI 1640 with 10% FBS; for Pam3SCK4 positive controls) for 60 minutes. The FBS used in this study was not heat inactivated. Following protein adsorption, surfaces were gently washed with PBS (three times, 5 minutes) then used immediately for cell culture or proteomic experiments.

### 2.4 Primary macrophage isolation and treatment

All animal work was approved by the Queen’s University (Kingston, ON, Canada) UACC (AUP 2018-1849). Bone marrow isolations were performed on wildtype (C57BL/6J, WT, stock# 000664), TLR2 knockout (TLR2^-/-^, stock# 004650) (37), and MyD88 knockout (MyD88^-/-^, stock# 009088) (38) mice (Jackson Laboratories, Bar Harbour, ME) that were bred and raised under sterile conditions in the Queen’s University Animal Care Facility. Prior to bone marrow isolations, mouse genotype was confirmed by PCR using a 1% agarose gel, based on manufacturer’s recommended protocols. The hind legs were removed and cleaned of tissue, then the bone marrow was flushed from the femur and tibia with sterile PBS, and red blood cells were with lysed with ammonium chloride. The remaining bone marrow cells were incubated in RPMI media (RPMI 1640, Sigma-Aldrich) containing 20% L929 supernatant, 10% FBS, and 50 µg/mL gentamicin and allowed to differentiate for at least 7 days (39). Differentiated bone marrow derived macrophages (BMDM) were used on day 7 to 10 for all experiments. Each isolation pooled bone marrow from multiple mice (WT: 8 mice, TLR2^-/-^ & MyD88^-/-^: 6 mice) and equal numbers of male and female mice were used for each bone marrow isolation to account for differences in TLR expression of murine macrophages between sexes (40). Four separate bone marrow isolations (from different litters of mice) were performed for each mouse genotype, giving a total of 32 WT mice, 24 TLR2^-/-^ mice and 24 MyD88^-/-^ mice used for this study.

BMDMs were washed with PBS (Gibco, Waltham, MA) and detached by incubation in TrypLE™ (Gibco) at 37 °C for 10 minutes. Cells were counted, resuspended in assay media, and plated in triplicate at 2.6 x 10^5^ cells/cm^2^ in the prepared Teflon™ AF coated wells with adsorbed protein layers. Pam3CSK4 (150 ng/mL, Cat. No. tlrl-pms, purity ≥ 95% (UHPLC), Invivogen) was included as a positive control for TLR2 signaling. Cells were cultured under the above conditions for 24 hours, followed by supernatant collection and RNA isolation for downstream analysis.

### 2.5 Flow cytometry

After the differentiation period, BMDM viability and differentiation was confirmed using flow cytometry (41). Cells were washed with PBS and detached by incubation in TrypLE™ (Gibco) at 37 °C for 10 minutes. Cells were resuspended at approximately 2 x 10^6^ cells/100 μL in PBS and incubated in 10 µg/mL anti-mouse CD16/32 (TruStain fcX™; Biolegend) on ice for 10 minutes, followed by incubation with Zombie NIR® (cat. No. 423105, Biolegend, San Diego, CA), 500 ng/ml of anti-mouse F4/80 (cat. No. 123115, Biolegend) and 1.25 µg/mL anti-mouse CD11b (cat no. 101235, Biolegend) on ice protected from light for 20 minutes. Cells were washed three times with staining buffer (5% FBS), and then resuspended in PBS. Flow cytometry was performed using a Beckman Coulter Cytoflex machine. Dead cells and cellular debris were gated out using a cell viability dye (Zombie NIR®, Biolegend), and an unstained control was used to confirm successful cell staining.

### 2.6 Quantitative PCR

RNA was collected from BMDMs after being cultured for 24 hours on Teflon™ AF surfaces using the RNeasy® mini kit (Qiagen, Hilden, Germany) according to manufacturer’s instructions. RNA was eluted in 30 µL of TE buffer and stored at -80 °C for future use. RNA concentrations and purity were measured using a NanoDrop One Spectrophotometer (Thermo Fisher Scientific, Waltham, MA) and all RNA samples had A260/A280 ≥ 1.8, A260/A230 ≥ 2.0. RNA quality was confirmed via non-denaturing agarose gel electrophoresis, by ensuring a 28S/18S intensity ratio of 2 or higher and no visible smear below the 18S band.

Isolated RNA was transcribed into cDNA using the iScript™ Reverse Transcription Supermix (BioRad) with 1 µg of RNA in each 20 μL reaction, according to manufacturer instructions. No reverse transcriptase (NRT) controls were made with RNA from WT BMDMs from each experimental condition (10% plasma, 10% lysate in plasma, Pam3CSK4) and run in a qPCR experiment to confirm there was no genomic DNA contamination after the RNA isolation procedure.

Specific murine primers for *Il10*, *Nos2*, and *Tnfα* were purchased from BioRad (*Il10*: qMmuCED0044967, *Nos2*: qMmuCID0023087, *Tnfα*: qMmuCEP0028054).The remaining primers were designed using PrimerBlast (42) and are listed in **Table 1**. qPCR was performed using SsoAdvanced Universal SYBR Green Supermix (BioRad), according to manufacturer instructions. The qPCR assay was run in a BioRad CFX384 system using 10 μL reactions in a 384 well plate, with 300 nM primers and 10 ng cDNA at 60 °C, with three biological replicates and conditions plated in quadruplicate. The relative gene expression ratio (R) was calculated using 2 reference genes (*Rplp0*, *Rpl13*), as described below (43). A plate of NRT controls was run to confirm there was no genomic DNA contamination in the RNA samples, and no amplification occurred in any NRT wells. No template controls (NTCs) were included in all assays (n = 3), and melt curves were performed at the end of every experiment to ensure no primer-dimers were formed.

**Table 1:** Primer sequences used in qPCR.

Gene	Accession Number	Forward Sequence (5’-3’)	Reverse Sequence (5’-3’)
Arg1	NM_007482.3	GTACATTGGCTTGCGAGACG	ATCGGCCTTTTCTTCCTTCCC
IL-1β	NM_008361.4	TGCCACCTTTTGACAGTGATG	ATGTGCTGCTGCGAGATTTG
IL-6	NM_031168	TAGTCCTTCCTACCCCAATTTCC	TTGGTCCTTAGCCACTCCTTC
MyD88	NM_010851.3	GAGGATATACTGAAGGAGCTGAAGTC	CCTGGTTCTGCTGCTTACCT
*Rpl13a	NM_009438.5	ATCCCTCCACCCTATGACAA	GCCCCAGGTAAGCAAACTT
*Rplp0	NM_007475.5	GGGCATCACCACGAAAATCTC	CTGCCGTTGTCAAACACCT
TGF-β1	NM_011577.2	AGCTGCGCTTGCAGAGATTA	AGCCCTGTATTCCGTCTCCT
TLR2	NM_011905.3	GGTGCGGACTGTTTCCTTCT	GAGATTTGACGCTTTGTCTGAGG
TLR4	NM_021297.3	TCCACTGGTTGCAGAAAATGC	TTAGGAACTACCTCTATGCAGGG

Arg1, Arginase 1; IL-1b, Interleukin 1 beta; IL-6, Interleukin 6; MyD88, Myeloid differentiation primary-response gene 88; Rpl13a, Ribosomal protein L13a; Rplp0, Ribosomal protein lateral stalk subunit P; TGF-β1, Transforming growth factor beta 1; TLR2, Toll-like receptor 2; TLR4, Toll-like receptor 4.

*Reference gene.

Data analysis of qPCR experiments was performed using a method described by Vandesompele et al. (43–45), which calculates the relative gene expression of each sample using the Ct values, and accounts for the use of two reference genes. Each biological replicate (n = 3, per experiment) was treated separately (46), and the geometric mean of the relative gene expression (R) was reported for each experiment (N = 4). Results were normalized to the 10% plasma condition (negative control) for each genotype. A two-way ANOVA of the log transformed normalized relative expression (NRE) was performed in GraphPad Prism 8.4.2 (GraphPad Software, San Diego, CA) to determine statistical difference in relative gene expression among treatment groups within a given genotype using an α = 0.05, as described by Taylor et al. (47). Changes in gene expression were only considered significant if median relative expression ratio (R) was less than 0.5 or greater than 2 (0.5 > R > 2) and the associated log_2_NRE p-value was less than 0.05.

### 2.7 Multiplexed bead-based cytokine assay

The supernatants of BMDMs cultured on Teflon™ AF-coated 6 well plates for 24 hours under the conditions of interest were collected, centrifuged at 1000 x g for 10 minutes to remove cellular debris, and stored at -80 °C for future analysis. The BMDM secretion of a variety of chemokines and cytokines was assessed using a Luminex assay (MilliPlex Magnetic 9-plex custom kit; MilliporeSigma, Burlington, MA), according to manufacturer directions. Samples were run undiluted in duplicate to measure the concentration of IL-1β, IL-6, IL-10, TNF-α, CXCL1 (keratinocyte chemoattractant, KC), CCL2 (monocyte chemotactic protein 1, MCP-1), CCL3 (macrophage inflammatory protein-1α, MIP-1 α), CCL5 (regulated upon activation, normal T cell expressed and secreted; RANTES), and vascular endothelial growth factor-A (VEGF-A) in the BMDM supernatant. Samples were plated across three 96 well plates, ensuring that samples from all conditions were present on each plate. On one plate the RANTES and VEGF-A samples did not pass the internal quality control, therefore, those points were excluded in the data analysis. Data was processed to obtain standard curves and cytokine concentrations using the BioPlex system (Ellis lab, Queen’s University; BioRad). Cytokine concentrations that were detectable but below the lowest standard were extrapolated and included in the data analysis. Non-detectable cytokine concentrations below the lower limit of detection excluded. Data points that were outside the lower and upper bounds of 1.5 times the interquartile range were considered outliers and excluded. Statistical analysis was performed in GraphPad Prism 8.4.3 (GraphPad Software, San Diego, CA) using a Brown-Forsythe and Welch ANOVA and Dunnett T3 *post-hoc* tests to determine significant differences among conditions. According to a power analysis, p < 0.05 was considered a statistically significant difference. Conditions that had less than 3 data points were not statistically analyzed. Results are displayed as mean ± SD, unless otherwise stated.

### 2.8 Proteomic analysis of adsorbed protein layers

#### 2.8.1 Liquid chromatography-tandem mass spectroscopy

Teflon™ AF coated well plates pre-conditioned with 10% plasma (with citrate) or 10% lysate in plasma were stored at 4 °C with 2 ml PBS/well overnight and then shipped on ice to the SPARC BioCentre (Molecular Analysis) at the Hospital for Sick Children (Toronto, Canada). The adsorbed protein from media containing 10% FBS was not analyzed. Proteomic samples were prepared using suspension trapping or S-trap high recovery method by the Sparc BioCenter. Briefly, adsorbed proteins from duplicate wells were scrapped into 8M urea, 5% sodium dodecyl sulfate (SDS) in 50 mM triethylamonium bicarbonate (TEAB) at pH 7.55 (50 μL). Samples were reduced using 4.6 mM tris carboxy ethyl phosphene (TCEP) at 37 °C for 15 minutes, then alkylated using 18.5 mM iodoacetamide in the dark for 30 minutes. Samples were loaded on to the S-Trap column (Protifi, Farmingdale, NY, USA) and digested on-column using 2.5 μg trypsin (Pierce) at 47 °C for 2 hours. Peptides were then eluted from the S-Trap column using four stepwise buffers: (1) 50mM TEAB (pH 8.0); (2) 0.1% formic acid; (3) 50% acetonitrile, 0.2% formic acid; and (4) 80% acetonitrile, 0.2% formic acid. The peptide solutions were lyophilized using a Speedvac, and resuspended in 2% acetonitrile, 0.1% formic acid.

Liquid chromatography–tandem mass spectrometry (LC–MS/MS) analysis was performed using an EASY-nLC 1200 nano-LC system coupled to a Orbitrap Fusion Lumos (Thermo Scientific). Peptides (1 μg peptide per sample) were loaded onto a PepMax RSLC EASY-Spray column (Thermo, 75 µm x 50 cm filled with 2 µM C18 beads; 900 Bar, 60°C) and separated over a 60-minute gradient of 3-35% organic phase (0.1% formic acid in acetonitrile) at 250 nl/min. Peptides were then analyzed using the Orbitrap Fusion Lumos mass spectrometer operating at 120 000 resolution over a mass range of m/z 375-1500. The raw data was searched against the mouse protein sequence database (Uniprot_UP000000589_Mouse_15092020.fasta) using Thermo Scientific Proteome Discoverer software (version 2.5.0.400).

#### 2.8.2 Proteomics analysis and protein classification

The database search results were imported to Scaffold (Scaffold_5.1.2) and the protein intensities, normalized on total precursor intensities, were retrieved. Proteins with FDR < 0.01 and covered by at least 2 peptides (FDR< 0.01) were retained for downstream analysis. Missing values were imputed by random sampling from the 1st percentile of all data distribution.

Differential protein expression analysis was calculated by moderate t-test using limma r package (48) on the log2 transformed protein intensities. A multiple testing adjusted p-value < 0.05 was considered significant (49). Gene set enrichment analysis (GSEA) was performed with the r package fgsea (50) (minsize = 2, maxsize = 500) on the ranked t-statistic, using the mouse gene ontology (GO) terms from the Molecular Signature Database (MSigDB) (51). GO enrichment was calculated for proteins significantly upregulated in Pla versus LysPla by a fold-change greater than 2; and proteins significantly upregulated in LysPla versus Pla by a fold-change greater than 2, by the gprofiler r package (52, 53) (organism = “mmusculus”, ordered_query = FALSE, exclude_iea = TRUE, user_threshold = 0.05, correction method = “g_SCS”, domain_scope = “annotated”).

A list of protein DAMPs was compiled from literature (**Supplemental Table 1**) and the list of proteins with differential expression (adjusted p-value < 0.05) was manually searched for known DAMP species in Excel (Microsoft) using the search terms listed. Identified DAMPs were highlighted in a volcano plot of the log2 foldchange (Pla vs LysPla) vs log(adjusted p-value) generated in GraphPad Prism. The mass spectrometry proteomics data have been deposited to the ProteomeXchange Consortium via the PRIDE (54) partner repository with the dataset identifier PXD042730.

## 3 Results

Prior to bone marrow isolation, mice were genotyped using TLR2 and MyD88 primers to confirm knockout genotype (**Supplemental Figure 1**). BMDM from WT, TLR2^-/-^ and MyD88^-/-^ bone marrow isolates were successfully differentiated into macrophages, with more than 93.5% of the populations staining positive for F4/80 and CD11b (**Supplemental Figure 2**). Representative light microscopy images showing BMDM morphology for each mouse strain and three culture conditions are provided in **Supplemental Figure 3**.

### 3.1 Gene expression of macrophages on Teflon™ AF

We first quantified the gene expression profile of cytokines and growth factors with well-documented roles in the progression of biomaterial host responses to study the effect of the adsorbed protein layer derived from 10% lysate in plasma on WT, TLR2^-/-^ and MyD88^-/-^ BMDM, compared to adsorbed protein layers derived only from plasma and soluble TLR2 agonist, Pam3CSK4. The mRNA expression of pro-inflammatory cytokines (IL-1β, IL-6 and TNF-α), anti-inflammatory cytokine IL-10 and pro-fibrotic growth factor TGF-β1 was quantified after 24 hours.

**Figure 1:**
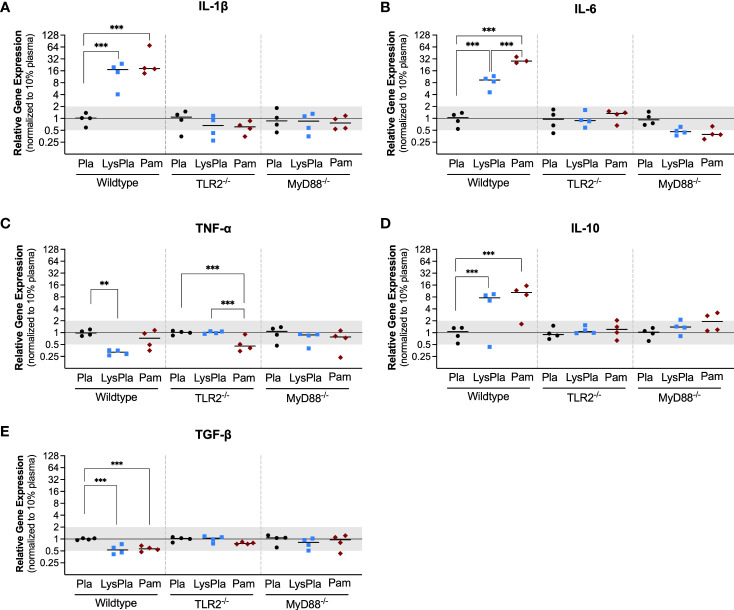
Relative gene expression of cytokines IL-1β **(A)**, IL-6 **(B)**, TNF-α **(C)**, IL-10 **(D)** and TGF-β **(E)** in WT, TLR2-/-, and MyD88-/- BMDMs cultured on Teflon™ AF for 24 hours. Each point represents the mean result of one experiment, where each condition had three biological replicates and was plated in triplicate for the qPCR assay. Results are displayed as median (bar) and individual (points) mean relative gene expression for each experiment. Pla, adsorbed 10% plasma (negative control); LysPla, adsorbed 10% lysate in plasma; Pam, Pam3CSK4 (TLR2 positive control). A two-way ANOVA of the log transformed NRE was used to determine statistical difference in relative gene expression among treatment groups within a given genotype, using an α = 0.05. Changes in gene expression were considered significant if the median relative expression ratio was less than 0.5 or greater than 2 (0.5 > R > 2) and the associated log2NRE p-value was less than 0.05, compared to 10% plasma within the same genotype. **p < 0.01 or ***p < 0.001 for associated log2NRE values.

The expression of genes encoding cytokines IL-1β, IL-6 and IL-10 was significantly upregulated in WT BMDMs exposed to adsorbed 10% lysate in plasma (LysPla) and Pam3CSK4 (Pam), compared to adsorbed 10% plasma (Pla) after 24 hours (**Figures 1A, B, D**). The effect of lysate and Pam3CSK4 was lost in TLR2^-/-^ and MyD88^-/-^ BMDMs, where gene expression appeared to be similar or slightly downregulated compared to 10% plasma, though these differences failed to show a statistically significant effect. Conversely, TNF-α mRNA expression was downregulated approximately 3-fold in WT BMDMs on adsorbed lysate at 24 hours (R = 0.32, p < 0.01 for LysPla vs Pla), while adsorbed lysate had no effect in the TLR2^-/-^ or MyD88^-/-^ BMDM (**Figure 1C**). Treatment with Pam3CSK4 caused a two-fold downregulation in TNF-α expression at 24 hours for the TLR2^-/-^ BMDM (R = 0.48, p < 0.001 for Pam vs Pla), but had no effect at the mRNA level for the WT or MyD88^-/-^ BMDM. The expression of TGF-β was also slightly downregulated at 24 hours in the WT BMDM for the lysate and Pam3CSK4 conditions (R = 0.53 and 0.57 respectively, p < 0.001 vs Pla), but was not considered to be biologically relevant as R > 0.05. No modulation of TGF-β expression was observed for any conditions in the TLR2^-/-^ or MyD88^-/-^ BMDM (**Figure 1E**).

**Figure 2:**
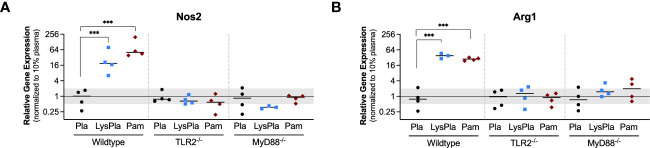
Relative gene expression of metabolic enzymes Nos1 **(A)** and Arg1 **(B)** in WT, TLR2-/-, and MyD88-/- BMDMs cultured on Teflon™ AF for 24 hours. Each point represents the mean result of one experiment, where each condition had three biological replicates and was plated in triplicate for the qPCR assay. Results are displayed as mean ± SD, with individual points showing the mean relative gene expression for each experiment. Pla, adsorbed 10% plasma (negative control); LyPla, adsorbed 10% lysate in plasma; Pam, Pam3CSK4 (TLR2 positive control). A two-way ANOVA of the log transformed NRE was used to determine statistical difference in relative gene expression among treatment groups within a given genotype, using an α = 0.05. Changes in gene expression were considered significant if the median relative expression ratio was less than 0.5 or greater than 2 (0.5 > R > 2) and the associated log2NRE p-value was less than 0.05, compared to 10% plasma within the same genotype. *** p < 0.001 for associated log2NRE values.

We next looked at the expression of genes encoding enzymes nitric oxide synthase 2 (Nos2) and Arginase 1 (Arg1) to gain insight into macrophage arginine metabolism (**Figure 2**) (55). Relative to BMDM cultured on adsorbates derived from 10% plasma, WT BMDM cultured on lysate-containing adsorbates had increased expression of both Nos2 and Arg1 (R = 18.9 and 37.9, p < 0.001, respectively). Treatment with Pam3CSK4 yielded a 50.5-fold increase in Nos2 expression and 27.9-fold increase in Arg1 expression, compared to 10% plasma (p < 0.001). BMDM derived from TLR2-deficient and MyD88-deficient mice did not have a significant response in Nos2 or Arg1 at the gene expression level following 24 hours of culture (0.5 < R < 2 and/or p-value of log_2_NRE > 0.05 compared to 10% plasma).

**Figure 3:**
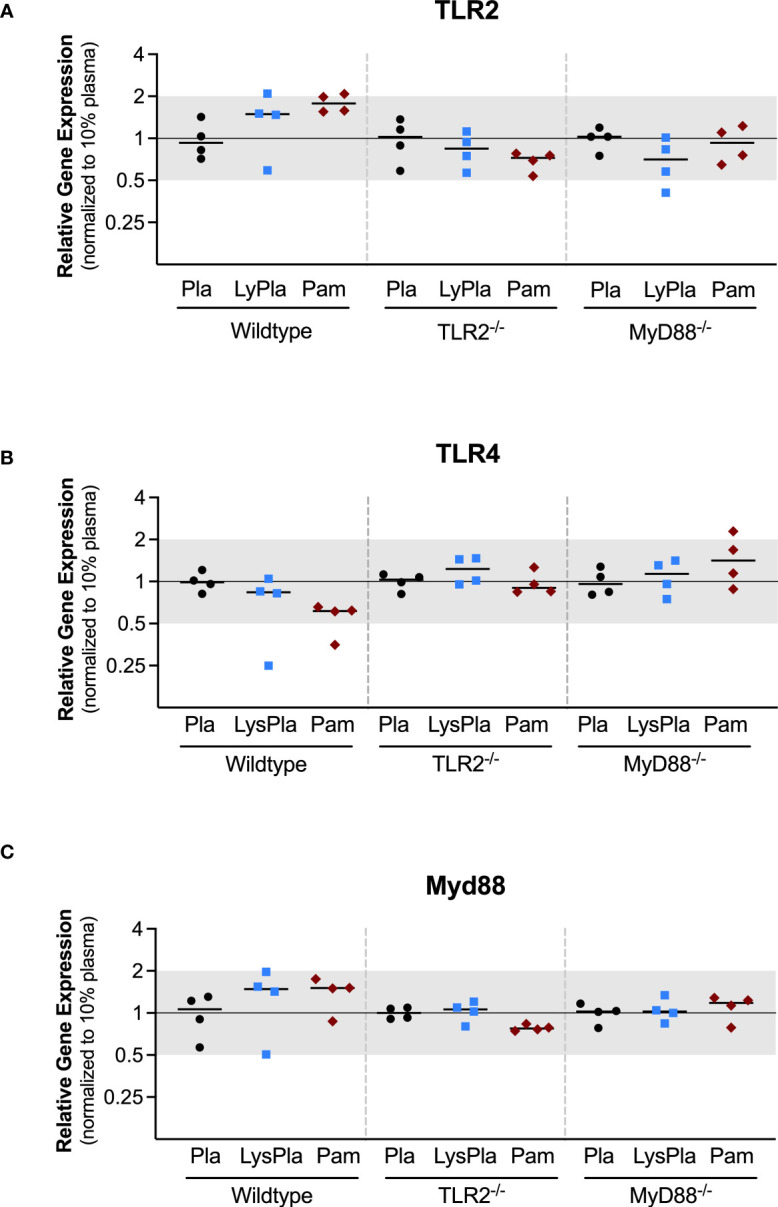
Relative expression of genes encoding TLR proteins TLR2 **(A)**, TLR4 **(B)**, and Myd88 **(C)** in WT, TLR2-/-, and MyD88-/- BMDMs cultured on Teflon™ AF for 24 hours. Each point represents the mean result of one experiment, where each condition had three biological replicates and was plated in triplicate for the qPCR assay. Results are displayed as mean ± SD, with individual points showing the mean relative gene expression for each experiment. Pla, adsorbed 10% plasma (negative control); LyPla, adsorbed 10% lysate in plasma; Pam, Pam3CSK4 (TLR2 positive control). * p < 0.05 compared to 10% plasma within the same genotype. A two-way ANOVA of the log transformed NRE was used to determine statistical difference in relative gene expression among treatment groups within a given genotype, using an α = 0.05. Changes in gene expression were considered significant if median relative expression ratio was less than 0.5 or greater than 2 (0.5 > R > 2) and the associated log2NRE p-value was less than 0.05, compared to 10% plasma within the same genotype.

The expression of genes encoding TLR2, TLR4, and Myd88 were also analyzed to study the influence of the TLR2 and MyD88 knockouts, which were created using targeted mutations at the genomic level, meaning the genes are still encoded in the mRNA but not made into functional proteins (**Figure 3**) (37, 38). No significant changes in the normalized gene expression of TLR2 or MyD88 were observed for any conditions or mouse strains (i.e., 0.5 < R < 2 and/or p-value of log_2_NRE > 0.05).

### 3.2 Cytokine production of macrophages on Teflon™ AF

**Figure 4:**
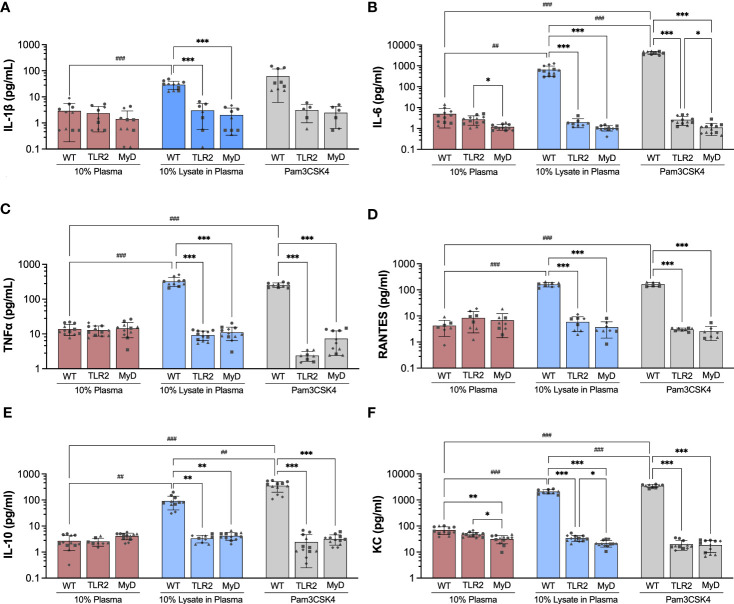
Concentration of IL-1β **(A)**, IL-6 **(B)**, TNF-α **(C)**, RANTES **(D)**, IL-10 **(E)** and KC **(F)** in supernatant of wildtype (WT), TLR2-/- (TLR2), and MyD88-/- (MyD) BMDMs cultured for 24 hours on Teflon™ AF with adsorbed 10% plasma (red bar), 10% lysate in plasma (blue bar) or with Pam3CSK4 (grey bar). Results are displayed as mean ± SD for four independent experiments (symbols ●, ■.◆, ▲ indicating experiment 1, 2, 3 and 4, respectively), each containing 3 replicates (n = 12). A Brown-Forsythe and Welch ANOVA and Dunnett T3 post-hoc tests were used to determine significant differences among conditions, with α = 0.05. ^##^p < 0.01 and ^###^p < 0.001 compared among WT groups. *p < 0.05, **p < 0.01 and ***p < 0.001 compared to WT within a treatment group.

The production of pro-inflammatory (IL-1β, IL-6, MCP-1, MIP-1α, RANTES/CCL5, TNF-α, KC/CXCL1), anti-inflammatory (IL-10), and angiogenic (VEGF-A) factors by BMDMs cultured on Teflon™ AF for 24 hours was assessed using a multiplexed bead-based cytokine assay (MilliPlex®, MilliporeSigma). In WT BMDM, exposure to adsorbed lysate significantly increased the secretion of pro-inflammatory cytokines and chemokines (IL-1β: 10.1-fold, IL-6: 128.8-fold, RANTES/CCL5: 28.9-fold, TNF-α: 23.2-fold, KC: 30.0-fold; p < 0.01) and anti-inflammatory cytokine IL-10 (33.63-fold, p < 0.01), when compared to adsorbed plasma (**Figure 4**; please refer to **Supplemental Figure 4** to view only WT data). Similarly, treatment with soluble Pam3CSK4 also increased cytokine secretion (IL-6: 837.4-fold, RANTES/CCL5: 66.4-fold, TNF-α: 18.4-fold, IL-10: 130.5-fold, KC/CXCL1: 49.7-fold, p < 0.001), although the increased expression of IL-1β (24.36-fold) was not statistically significant (p > 0.05) due to high variability in IL-1β expression in the Pam3CSK4 group. VEGF-A was detected in the WT BMDM supernatants, however no differences in concentration were observed among the three treatments (p > 0.05, **Supplemental Figure 4H**). Exposure to the adsorbed lysate condition appeared to have a similar effect on WT BMDM cytokine secretion when compared to the soluble TLR2 agonist, although Pam3CSK4 treatment did yield higher concentrations of IL-6, KC, and IL-10 (p < 0.01 for WT LysPla vs WT Pam). No significant differences were observed for MIP-1α, MCP-1 or VEGF for WT BMDM cultured on 10% plasma, 10% lysate in plasma or with Pam3CSK4 (**Supplemental Figures 4E, F, H**).

Next, we focused on the effect of TLR2 and MyD88 on the concentration of cytokines that were increased in WT BMDM in response to lysate or Pam3CSK4 conditions. When cultured on Teflon™ AF pre-conditioned with 10% plasma, BMDM mouse strain (WT, TLR2-/-, MyD88-/-) had no effect on the concentration of cytokines (p > 0.05), except for IL-6 and KC (**Figure 4**). While no difference in IL-6 concentration was observed between WT and TLR2^-/-^ BMDM on plasma-adsorbed surfaces, IL-6 was reduced in the MyD88^-/-^ BMDM compared to TLR2^-/-^ BMDM (p < 0.05, **Figure 4B**). The supernatant concentration of KC was lower for TLR2^-/-^ and MyD88^-/-^ BMDM on adsorbed plasma, compared to the WT BMDM on plasma (p < 0.05) (**Figure 4F**).

In contrast to WT macrophage, exposure to the adsorbed lysate did not elicit an increase in cytokine production in TLR2-deficient and MyD88-deficient macrophages (p > 0.05 for compared to the 10% plasma), supporting our earlier findings in RAW264.7 and RAW-Blue macrophages that TLR2 was the main mediator of macrophage activation in response to adsorbed lysate (28, 29). Furthermore, WT BMDM had significantly higher concentrations of cytokines (IL-1β, IL-6, TNF-α, RANTES, IL-10 and KC) on lysate-containing adsorbates, compared to TLR2^-/-^ and MyD88^-/-^ BMDM. As expected, the TLR2^-/-^ and MyD88^-/-^ BMDM also failed to respond to Pam3CSK4 stimulation (p > 0.05, compared to 10% plasma; comparisons not shown in **Figure 4**), with the exception that TLR2^-/-^ BMDM had decreased TNF-α (5.4-fold decrease, p < 0.05) and KC (2.4-fold decrease, p <0.001) expression compared to 10% plasma. No differences in cytokine concentrations were found between TLR2 and MyD88 knockout macrophages for any conditions; except for IL-6 and KC. IL-6 was lower in MyD88^-/-^ supernatants, compared to TLR2^-/-^ for Pam3CSK4 (p < 0.05, **Figure 4B**), while KC concentrations were lower in MyD88^-/-^ supernatants compared to TLR2^-/-^ for 10% plasma and 10% lysate in plasma (p < 0.05, **Figure 4F**).

### 3.3 Proteomic analysis of adsorbed protein layers on Teflon™ AF surfaces

**Figure 5:**
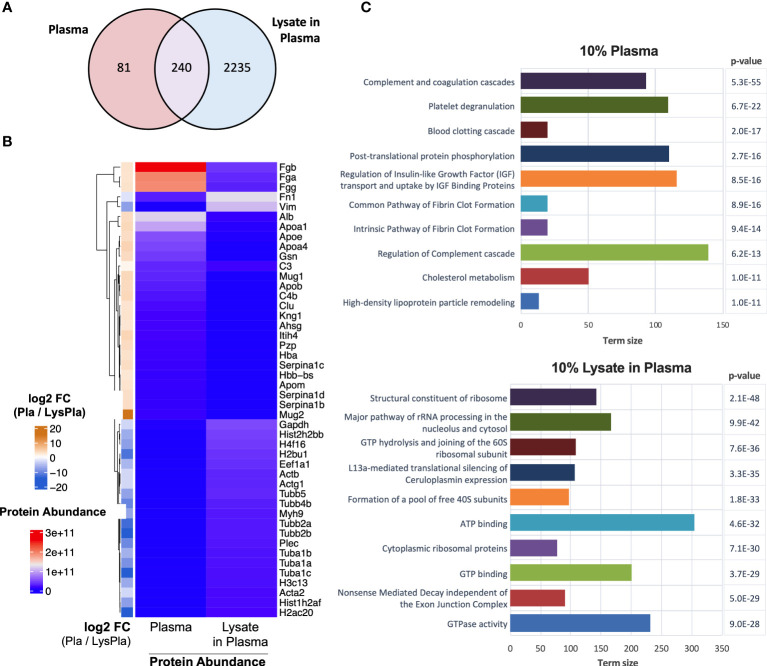
Proteomic analysis of adsorbed proteins layers by LC-MS/MS. **(A)** Venn diagram of identified proteins in adsorbates derived from 10% plasma and 10% lysate in plasma on Teflon™ AF surfaces. **(B)** Heat map of top 25 abundant proteins identified in adsorbed protein layers derived from 10% plasma and 10% lysate in Plasma adsorbed proteins with corresponding log2 fold-change for plasma vs lysate in plasma. **(C)** The top ten GO terms enriched in differentially expressed (fold-change > 2) in either the 10% plasma (upper) or 10% lysate in plasma (lower) conditions. Differential protein expression analysis was calculated by moderate t-test using limma r package (48) on the log2 transformed protein intensities. A multiple testing adjusted p-value < 0.05 was considered significant (49).

Next, we analyzed the composition of the adsorbed protein layers generated from 10% plasma and 10% lysate in plasma using LC-MS/MS to determine whether the potent TLR2-dependent macrophage activation on the lysate-adsorbed surfaces was associated with the presence of known DAMPs within the adsorbed protein layer. The proteomic analysis identified 321 proteins in the adsorbed layers derived from 10% plasma, while 2556 were identified in the adsorbed layers derived from the 10% lysate in plasma mixtures (**Figure 5A**). To better understand how a 10% w/w spike of lysate affected the adsorption of plasma proteins, we then compared the 25 proteins with the highest log2 transformed protein intensities in each condition (**Figure 5B**). The 10% plasma condition yielded a list of proteins that included many of the well-studied proteins in adsorption literature, including fibrinogen (Fgb, Fga, Fgg), albumin (Alb), apolipoproteins (Apoa1, Apoe, Apoa4, Apob, Apom), complement (C3, C4), fibronectin (Fn1), and kininogen 1 (Kng1). Proteins layers generated from plasma that contained a 10% w/w spike of lysate proteins retained fibrinogen (Fgb, Fgg, Fga) and fibronectin (Fn1) remained among the top 25 proteins. However, these lysate-containing adsorbed protein layers had a disproportionate increase in intracellular proteins, including histones (H2bu1, H3c13, H4f16, Hist1h2af, Hist2h2bb), tubulins (Tuba1a, Tuba1b, Tuba1c, Tubb2a, Tubb2b, Tubb4b, Tubb5), actins (Acta2, Actb, Actg1), vimentin (Vim) and myosin 9 (Myh9). The full list of identified proteins in both adsorbed proteins layers and their relative intensities is provided in **Supplemental Data File 1**.

We then identified proteins that were enriched in either condition using a moderate T-test on the log2 transformed protein intensities, where a multiple testing adjusted (Benjamini) p-value < 0.05 and fold-change > 2 in absolute value were considered significant. A complete list of significantly enriched proteins with their fold-change and adjusted p-value are provided in **Supplemental Data File 2**. Functional enrichment analysis was calculated for all proteins differentially expressed in either the 10% plasma or 10% lysate in plasma conditions (**Supplemental Data File 3**) and the top ten enriched pathways for each adsorbed protein layer are shown in **Figure 5C**.

**Figure 6:**
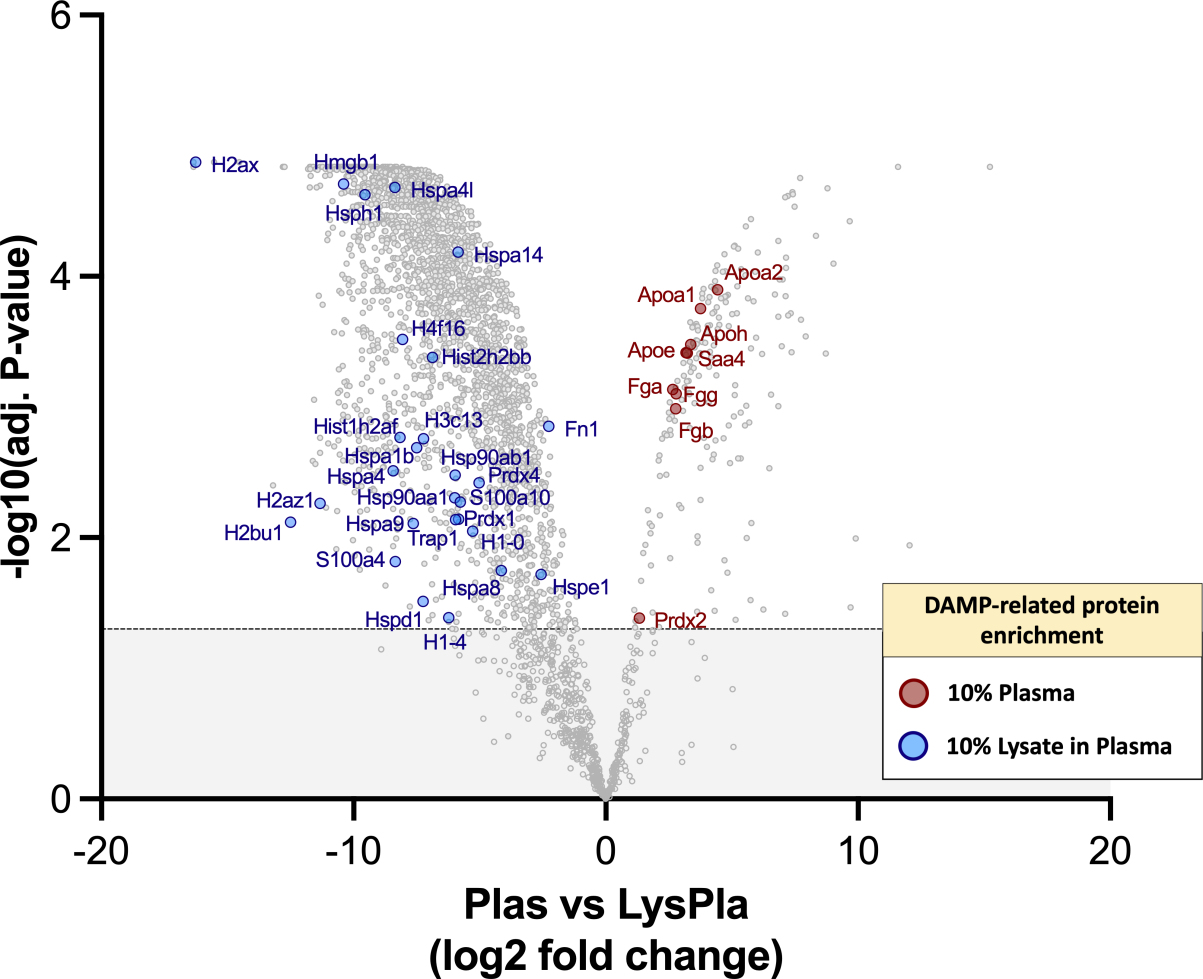
DAMPs identified with adsorbed protein layers on Teflon™ AF surfaces following 1 hr incubation at 37 oC in 10% plasma or 10% (w/w) lysate in plasma. Volcano plot representing the statistical analysis of the normalized proteins intensities in plasma samples versus lysate in plasma samples, with DAMPs labeled. The x-axis shows the log2 foldchange of each identified protein and the y-axis the corresponding −log10 P value. Proteins with adjusted p-values greater than 0.05 (shown as dotted line) were considered significant.

Finally, we sought to determine if the 10% lysate in plasma protein layer was enriched for DAMPs, which may account for the increased TLR2-/MyD88-dependent macrophage activation observed on these surfaces. However, DAMPs, to our knowledge, currently do not have a specific annotation with the gene ontology databases we consulted. Therefore, a manual approach was required. We first compiled a list of putative protein DAMPs from published literature (**Supplementary Table 1**) and then manually searched the protein lists for protein or protein classes reported as DAMPs within literature. Using this approach, we identified 39 DAMPs or DAMP-related proteins enriched in the 10% lysate in plasma condition, compared to seven in the 10% plasma condition (**Figure 6**). A caveat to this manual approach is that the list does not represent an exhaustive list of all putative DAMPs within the literature, and therefore, we view these results as hypothesis, rather than conclusion, generating.

## 4 Discussion

Within the field of biomaterials, it is well-established that the adsorbed protein layer on an implanted biomaterial surface mediates cell-material interactions and the progression of the host response. The adsorption of blood proteins has been studied extensively for more than fifty years (11, 13, 20) and provides a strong foundation for understanding cell-material interactions. During biomaterial implantation, damage to the local tissue would lead not only to blood leakage from damaged vasculature, but also the release of DAMPs from damage extracellular matrix and cells within the implant site (27). However, little is understood regarding the adsorption of non-blood derived proteins or other types of molecules. In 2018, our group first demonstrated that molecules within 3T3 fibroblast lysates adsorb to biomaterial surfaces and potently activate macrophages in a TLR2-dependent manner (28), even in the presence of serum proteins. We went on to demonstrate that the cytokine profile of RAW264.7 macrophages, a mouse macrophage cell line, over 5 days recapitulated the cytokine profile reported during *in vivo* macrophage-material interactions, induced low rates of macrophage fusion and promoted the late expression of pro-fibrotic TGF-β1 (29). However, these studies were limited by the use cell lines and protein layers derived from 100% fibroblast lysate. Furthermore, no characterization of the adsorbed protein layers was conducted to understand what types of molecules were adsorbing to the biomaterial surfaces. In the present study, we address these limitations by using primary mouse macrophages and adsorbed protein layers derived from either mouse plasma or plasma spiked with lysate (10 w/w%) to better model *in vivo* protein adsorption, and lysate adsorption in competition with plasma proteins. Furthermore, we further characterized the relative importance of TLR2 and TLR adapter protein, MyD88, in the response to adsorbates containing lysate-derived molecules using macrophage-derived from knockout mice. The study aimed to elucidate the ability of lysate-derived molecules to adsorb onto Teflon™ AF surfaces in the presence of blood proteins and activate primary mouse macrophages. To this end, K2 EDTA and citrated plasma preparations were used to eliminate the effect of the complement cascade (56–59), which is recognized as an important factor in biomaterial host responses (56, 58, 60, 61). As 10% FBS was used, some degree of complement activation was possible in all samples. However, our previous work found that adsorbed protein layers derived from 10% FBS, 10% plasma and 10% heat-inactivated FBS yielded similar, minimal NF-κB/AP-1 activity in mouse reporter macrophages, supporting our assumption that complement activation was negligible in this model, compared to the effect of the adsorbed lysate (30).

In the present study, we demonstrated that adsorbed protein layers derived from mouse plasma spiked with small amounts of mouse lysate (10% w/w total protein) induced a potent pro-inflammatory response in primary mouse macrophages at 24 hours. This response was characterized by increased gene expression and cytokine concentration of acute phase cytokines (IL-1β, IL-6, TNF-α, IL-10) and chemokines (RANTES/CCL5, KC/CXCL1), and was dependent on TLR2/MyD88 signaling. This increased cytokine and chemokine expression was consistent with reported cytokine expression within *in vivo* implant sites during the early phase (e.g. day 4 and 7) of the FBR to synthetic biomaterial implants (62).

While the gene and protein expression data showed consistent trends for most factors, a notable exception was TNF-α. While the secreted protein concentration was significantly elevated (~ 60-fold increase compared to 10% plasma), the relative gene expression at 24 hours was similar or slightly downregulated in the lysate condition, compared to plasma. Baer and colleagues have shown a similar trend in TNF-α gene expression of BMDMs exposed to LPS over time, where up to 3 hours post-exposure the mRNA expression of TNF-α increased, and then over time decreased to baseline levels (63). Their work, as well as the work here, demonstrates that TNF-α is an early response cytokine, and over time it can become downregulated as other pro-inflammatory and anti-inflammatory cytokines are produced (63). Baer et al. and others have shown that the NF-κB p50 subunit is responsible for the downregulation of TNF-α in murine and human primary macrophages (63, 64). Alexander et al. have also demonstrated that in mouse macrophages TNF-α is regulated by IL-10 (65). Therefore, the upregulation of IL-10 secretion in WT BMDMs exposed to lysate is a likely contributing factor to the downregulation of TNF-α gene expression in these macrophages.

The gene expression of metabolic enzymes Nos2 and Arg1 was compared to gain insight into the metabolic state and polarization of lysate-stimulated macrophages (55). Lysate-stimulated WT macrophages upregulated the gene expression of both enzymes, with Arg1 having a slightly higher fold-increase compared to Nos2, relative to the macrophages on plasma-adsorbed Teflon™ AF. Although Nos2 and Arg1 are typically regarded as distinct metabolic markers of M1 and M2 macrophage phenotypes, there are several reports of macrophages expressing both enzymes under specific conditions, including a TLR-dependent response to mycobacterium that induced Arg1 expression (66). Considering the two extremes of macrophage polarization, the WT macrophage population stimulated by adsorbed lysate or Pam3CSK4 appear to lie on the spectrum between a classical M1 macrophage phenotype characterized by high IL-6, TNF-α, Nos2 and an alternative M2 macrophage phenotype with high IL-10 and Arg1 expression. This cytokine profile is consistent with previous reports that Pam3CSK4 induced an immunosuppressive M2-like macrophages from human monocytes that express both IL-10 and IL-6, which makes Pam3CSK4 unique among TLR agonists that generally induce an M1 phenotype (67–69). Similarly, others have shown that Pam3CSK4 induced human monocytes to produce IL-1β and IL-6 via canonical (p65/RelA) NF-κB signaling pathway and IL-10 via the non-canonical (p100/p52) pathway (70). However, the cytokine and gene expression data reflect the global population and lack the robust selection of immunophenotyping markers required for macrophage polarization analysis. Therefore, we cannot determine whether the lysate-containing adsorbates promote a similar immunosuppressive M2-like phenotype as with Pam3CSK4 (although the profile are highly similar), a mixture of M1 and M2 macrophage populations, or a hybrid M1/M2 macrophage phenotype that has been observed *in vivo* at implant sites (71–73). Regardless, the induction of both pro-inflammatory and anti-inflammatory mediators may reflect the fact that the adsorbates derived from lysate and plasma contained a multitude of potential stimuli or activate macrophages via a similar signaling mechanism as Pam3CSK4. Conversely, Teflon AF™ surfaces with adsorbates derived solely from mouse plasma yielded low (~ 10 pg/ml or lower) expression of many of the cytokines associated with polarization and/or FBR (e.g., IL-1β, IL-6, TNF-α, IL-10), suggesting adsorbed molecules derived from K2 EDTA plasma did not significantly activate the WT macrophages. This negligible activation on the plasma adsorbates was expected, as the calcium chelation by the plasma anticoagulant (K2 EDTA) inhibits activation of the complement cascade, which otherwise would also induce an inflammatory response (26). Chemokines MCP-1 and MIP-1α and angiogenic growth factor VEGF were expressed at higher concentrations ranging (~ 100 to 800 pg/ml) on all surfaces, indicating their expression was induced by the 10% plasma condition or a basal expression for BMDM on Teflon™ AF surfaces.

MyD88 is a critical adaptor protein for TLR2/1 and TLR2/6 heterodimers, but also all other TLR (except TLR3) as well as IL-1β signaling (74). Therefore, it was expected that the loss of the MyD88 protein may have a more robust effect on the macrophage response than the more selective loss of TLR2. However, TLR2 and MyD88 knockout macrophages yielded highly similar cytokine expression profiles at the protein and mRNA level in this model. This supports our original hypothesis that TLR2 is the primary TLR2 mediator of macrophage activation in response to Teflon™ AF surfaces pre-adsorbed with 10% lysate in plasma. These results are significant as they open the door for potential therapeutic strategies that target DAMP-induced macrophage activation in a broader (MyD88) or more selective (TLR2) manner.

Proteomic analysis of the adsorbed protein layers generated from 10% plasma or 10% lysate in plasma on the Teflon™ AF surfaces clearly demonstrated that even small amounts of cell-derived molecules in the presence of blood-derived molecules can significantly alter the adsorbed protein profile on Teflon™ AF surfaces. Teflon™ AF is a hydrophobic fluoropolymer that was used to model Teflon™ cannulas of IIS. Hydrophobic fluoropolymers are known to bind serum protein almost instantaneously and have high protein retention (75, 76). Here, Teflon™ AF surfaces pre-adsorbed with 10% plasma yielded proteomic profile abundant (in terms of protein intensities) in fibrinogen, albumin, apolipoproteins, complement proteins and fibronectin. This profile is consistent with previous proteomic studies of protein adsorption from heparinized plasma for other hydrophobic materials (61), as well as earlier work with Teflon™ (PTFE) using more traditional protein adsorption methodologies (75, 76). Protein adsorption from blood and blood products has been an active area of research in the biomaterials field for more than fifty years, and was recently summarized in a comprehensive series of reviews on blood-material interactions and its subsequent effects on biocompatibility (24, 60, 77–79).

Proteomic analysis of adsorbed protein layers generated *in vivo* is less well characterized. Swartzlander et al. used LC-MS/MS proteomic to characterize the protein layers adsorbed on hydrophilic polyethylene glycol (PEG) hydrogels following a 30-minute subcutaneous implantation in mice. In this study, albumin was the most abundant protein, while apolipoproteins, complement C3, murinoglobulin 1 (Mug1) were also among the “top 20” adsorbed proteins, similar to the 10% Plasma Teflon™ AF condition in the current study. Many of the other abundant proteins in the PEG hydrogels were associated with the acute inflammatory and wound healing processes, and located within the extracellular compartment (27). However, approximately 10% of the identified proteins came from the intracellular compartment, in particular the cytoskeleton and cytosol (27), suggesting that *in vivo* protein adsorption layers do acquire proteins released damaged cells in the surroundings.

We demonstrated that by adding 10% (w/w of total protein) lysate proteins to the plasma significantly altered the adsorbed protein profile on Teflon™ AF surfaces. Although fibrinogen and fibronectin remained in the top 25 proteins, many other blood proteins, including albumin (ranked 36^th^) and complement C3 (ranked 30^th^) were replaced primarily by the cytoskeletal proteins (actins, tubulins, vimentin, myosin) and histones. Significantly, the 10% lysate in plasma adsorbates were enriched for well characterized DAMPs high mobility group box 1 (HMGB1) and core histones (80), as well as putative DAMPs heat shock proteins (HSP70, HSP60) and S100 proteins (33). As the 10% lysate in plasma surfaces activated primary mouse macrophages in a TLR2/MyD88-dependent manner, we were particularly interested in DAMPs that are known to act via this pathway. HMGB1 is a nuclear chromatin-binding protein, but when released from cells via either active secretion or passive release in response to tissue damage, it mediates inflammation via its interaction with TLR4 and receptor for advanced glycation end-products (RAGE) (81). There are conflicting reports on the ability of HMGB1 to induce a cytokine response via TLR2. However, a recent study revealed that HMGB1 interacts with TLR2, but function in complex with other known TLR2 agonists to enhance TLR2 signaling (82). When released to the extracellular fluid in response to trauma or severe cellular stress, histones (H1, H2A, H2B, H3, and H4) signal through TLR2, TLR4 and TLR9 to induce the production of cytokines (e.g., IL-6, IL-10 TNF-α), activate the NALP3 inflammasome and complex with other DAMPs (e.g., DNA, HMGB1) to act as a co-activator (80, 83–85). With the adsorbed protein layer derived from 10% lysate in plasma, 16 proteins from the H1, H2A, H2B, H3 and H4 families were identified and enriched, compared to 10% plasma only. Extracellular peroxiredoxin-1 (Prdx-1) has been found to induce chemokine production via TLR2/4/MyD88 (KC/CXCL1, MIP-2α/CXCL2, MCP-1/CCL2) and TLR4/TRIFF (RANTES/CCL5) (86), and trigger sterile inflammation in models of acute injury, including ischemic brain injuries, acute liver injury and acute lung injury (87–90). We also noted the presence of putative DAMPs, such as HSP, which have conflicting evidence of true DAMP activity. While HSP are frequently cited as DAMPs, there is controversy within the literature regarding the role of extracellular HSP in immunity (91). While earlier work showed HSP acted as DAMPs via TLR, later studies suggest that this response was due, at least in part, to contaminants within the recombinant protein preparations (92).

### 4.1 Study limitations

The concentration of cytokines was reported per well and was not normalized to the number of cells in each condition. Therefore, it is possible that differences in reported cytokine concentrations among the experimental conditions may reflect differences in the number of BMDM. However, visual observations of the wells at 24 hours (representative images shown in **Supplemental Figure 1**) did not reveal a notable difference in cell density. As most cytokine concentrations differed by an order of magnitude or more between the wildtype condition and knockout strains, we do not anticipate that variations in cell density among samples (if present) would change the study conclusion that lysate-containing adsorbates stimulated macrophages in a TLR2/MyD88 dependent manner.

Collectively, the proteomic profile of the adsorbed protein layer on Teflon™ AF substrates pre-conditioned with 10% lysate in plasma provides multiple potential ligands that may elicit, either alone or in combination, a TLR2/MyD88-dependent macrophage response observed on lysate-derived adsorbates within this study, as well as our previous work with RAW-264.7 and RAW-Blue reporter macrophages. However, there are multiple limitations and caveats that must be acknowledged before drawing any definitive conclusions regarding the mechanisms of action of the adsorbed lysate model and its relevance to biomaterial host responses and the FBR. First, the proteomic method used to analyze the adsorbed protein layer generates a relative abundance of proteins present in each condition that does not linearly correlate with the actual protein copy numbers in each sample. Furthermore, these results will be influenced by multiple factors, including the number of tryptic peptides each protein generates after trypsin digestion, the ionizability of the peptides and other factors (24, 93). Therefore, the presence of proteins of interest require validation using other methods, such as immunological assays (ELISA, Western Blot) or targeted MS assays (93). Furthermore, immunodepletion or blocking of proteins of interest would be required to demonstrate the relative importance of that protein’s contribution to macrophage activation. Moving beyond validating the presence and function of different proteins within the adsorbed protein layers generated in the present model, there is the critical question of whether this model is useful and predictive of macrophage-material interactions *in vivo*. Our work supports previous studies by Stephanie Bryant and colleagues, who have demonstrated that MyD88-dependent signaling is a key regulator of inflammatory cell recruitment and fibrous capsule formation in PEG-hydrogel implant models *in vivo (* 32). Compared to the proteomic analysis of *in vivo* generated protein layers on PEG hydrogels, the 10% lysate in plasma was more enriched for intracellular proteins, suggesting that reducing the amount of lysate may result in a more accurate model of *in vivo* protein layers. However, PEG is a hydrophilic hydrogel and Teflon™ AF is a hydrophobic amorphous polymer, and the two materials differ in many properties that are known to influence protein adsorption. Another approach to improve the physiological relevance of this present model is to use an anticoagulant that preserves the complement and elements of the coagulation cascade. For example, the thrombin inhibitor lepirudin preserves the complement cascade and the coagulation cascade upstream of thrombin, making a more representative plasma model of the *in vivo* environment (94). Recent proteomic analysis of adsorbed protein layers from human lepirudin-plasma demonstrated distinct differences in the levels of complement and coagulation activators and inhibitors present on the surface of three types of alginate microsphere (26). As adsorbed complement components and activation of the alternative amplification loop on material surfaces are important factors in biomaterial host responses, the use of lepiruinated plasma with a lysate spike should be considered for the further development of *in vitro* protein adsorbption models related to biomaterial inflammatory responses (56–61, 95). Ultimately, a proteomic analysis of *in vivo*-generated adsorbed protein layers on Teflon™ AF surfaces would provide a more useful comparison to determine how well this *in vitro* model or future iterations recapitulate the *in vivo* scenario. Furthermore, *in vivo* studies exploring the response of TLR2- and MyD88-deficient mice to Teflon™ or Teflon™ AF implants are required to demonstrate the important of TLR2-signaling within the host response to the fluoropolymers, and other biomaterials. Finally, as the present and preceding studies of the effect of adsorbed lysate-derived molecules have focused on mouse macrophages, it is necessary to validate these findings using human macrophage models in future research.

## 5 Conclusion

In summary, our study provides evidence that adsorbed protein layers containing plasma and cell lysate activate primary bone-derived macrophages in a TLR2-dependent manner to express pro-inflammatory (IL-1β, IL-6, TNF-α, RANTES, Nos2) and anti-inflammatory or tolerizing factors (IL-10, Arg1). Proteomic profiling of the adsorbed layers from lysate-containing plasma solutions suggests that known intracellular DAMPs, such as HMGB1 and histones, are enriched on the surface of the Teflon™ AF, and that subsets of these proteins are known to induce sterile inflammatory responses through TLR2. Further studies will be required to validate the presence of these TLR2-binding DAMPs and their contribution to macrophage activation in the present *in vitro* model of macrophage-material interactions, as well as assess TLR2/MyD88-dependent signaling within *in vivo* biomaterial implant models. Overall, our study contributes to the growing body of evidence supporting TLRs as modulators of macrophage-material interactions and biomaterial host responses.

## Data availability statement

The proteomic datasets presented in this study can be found in online repositories. The names of the repository/repositories and accession number(s) can be found in the article/**Supplementary Material**. All other raw data supporting the conclusions of this article will be made available by the authors, without undue reservation.

## Ethics statement

The animal study was approved by University Animal Care Committee (UACC; AUP 2018-1849) at Queen’s University (Kingston, ON, Canada). The study was conducted in accordance with the local legislation and institutional requirements.

## Author contributions

LM and LF contributed to the conception, design of the study, formal analysis of the data. LM wrote the first draft of the manuscript. LM and LB performed the investigation, validation and contributed to the methodology. GN performed the proteomic data analysis and visualization, and writing (review and editing). KW and LF contributed resources, writing (review and editing), supervision and funding acquisition. All authors contributed to manuscript revision, read, and approved the submitted version.
