# Supplementary material for: MyD88-dependent Toll-like receptor 2 signaling modulates macrophage activation on lysate-adsorbed Teflon™ AF surfaces in an in vitro biomaterial host response model
Source: Front Immunol. 2023 Aug 24;14:1232586. doi: 10.3389/fimmu.2023.1232586 (PMC10491479; doi:10.3389/fimmu.2023.1232586)
Supplement: Supplementary file 4 [file DataSheet_4.docx]

Supplementary Material

MyD88-dependent Toll-like receptor 2 signalling modulates macrophage activation on Teflon™ AF surfaces in an in vitro biomaterial host response model

**Laura A. McKiel, Laurel L. Ballantyne, Gian Luca Negri, Kimberly A. Woodhouse, and Lindsay E. Fitzpatrick^*^**

*** Correspondence:** Corresponding Author: lindsay.fitzpatrick@queensu.ca

# Supplementary Data Files

Supplementary Data File 1 – Normalized Protein Intensities

Supplementary Data File 2 – Protein with Differential Expression (log2FC)

Supplementary Data File 3 – Functional Enrichment Analysis of Differentially Expressed Proteins

# Supplementary Figures and Tables


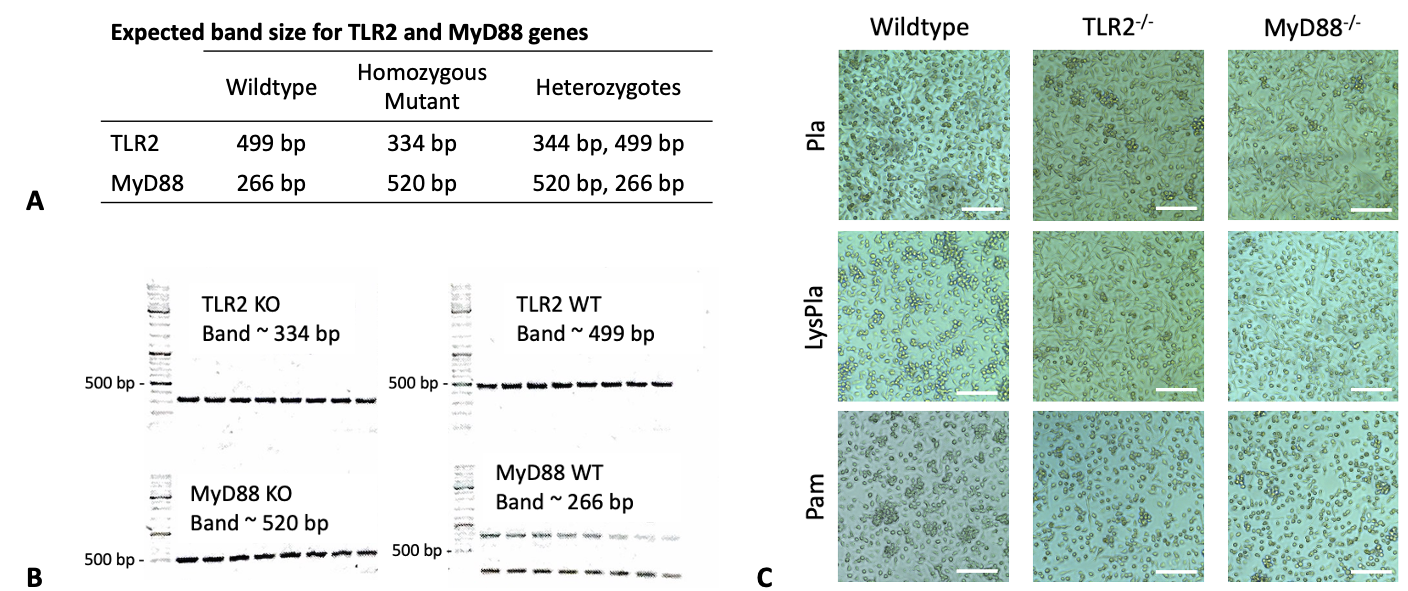


**Supplemental Figure 1.** (A) Expected band sizes for TLR2 and MyD88 genes from wildtype, homozygous mutants, and heterozygotes for TLR2 (JAX Stock No: 004650) and MyD88 (JAX stock# 009088) knockout mice. (B) Representative 1% agarose gels of TLR2 and MyD88 bands from wildtype, homozygous TLR2 mutants and homozygous MyD88 mutants, using a GeneRuler DNA Ladder Mix (SM0333, ThermoFisher).


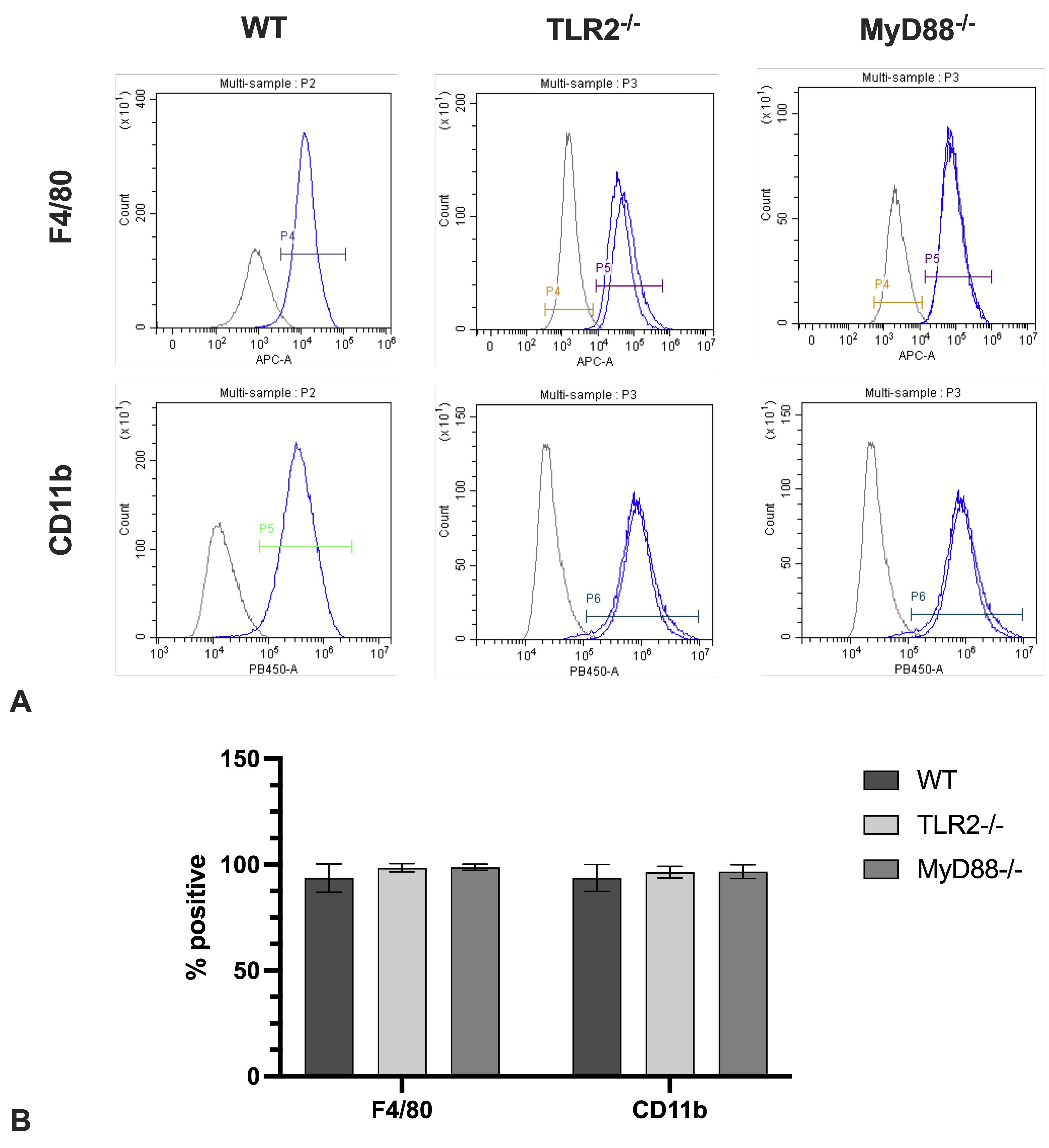


**Supplemental Figure 2.** (A) Example histograms of flow cytometric analysis of F4/80 and CD11b on BMDM populations from wildtype (WT), TLR2^-/-^ and MyD88^-/-^ mice following a 7-day differentiation. (A) Representative histograms for stained (blue histogram) BMDM and unstained (grey histogram) controls. (B) Percentage of BMDM staining positive for F4/80 and CD11b after differentiation. Data was analyzed using a one-way ANOVA. Mean +/- SD, n = 4.


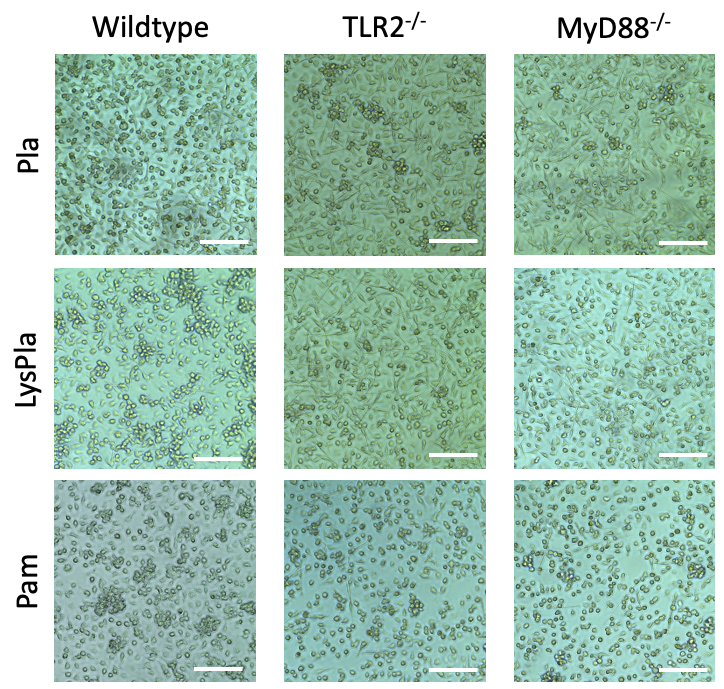


**Supplemental Figure 3.** Micrographs of BMDM macrophages isolated from wildtype, TLR2-/- and MyD88 -/- mice, following 24h culture on PTFE surfaces under the three culture conditions of 10% plasma (Pla), 10% lysate in plasma (LysPla) and Pam3CSK4. Scale bar represents 100 μm.


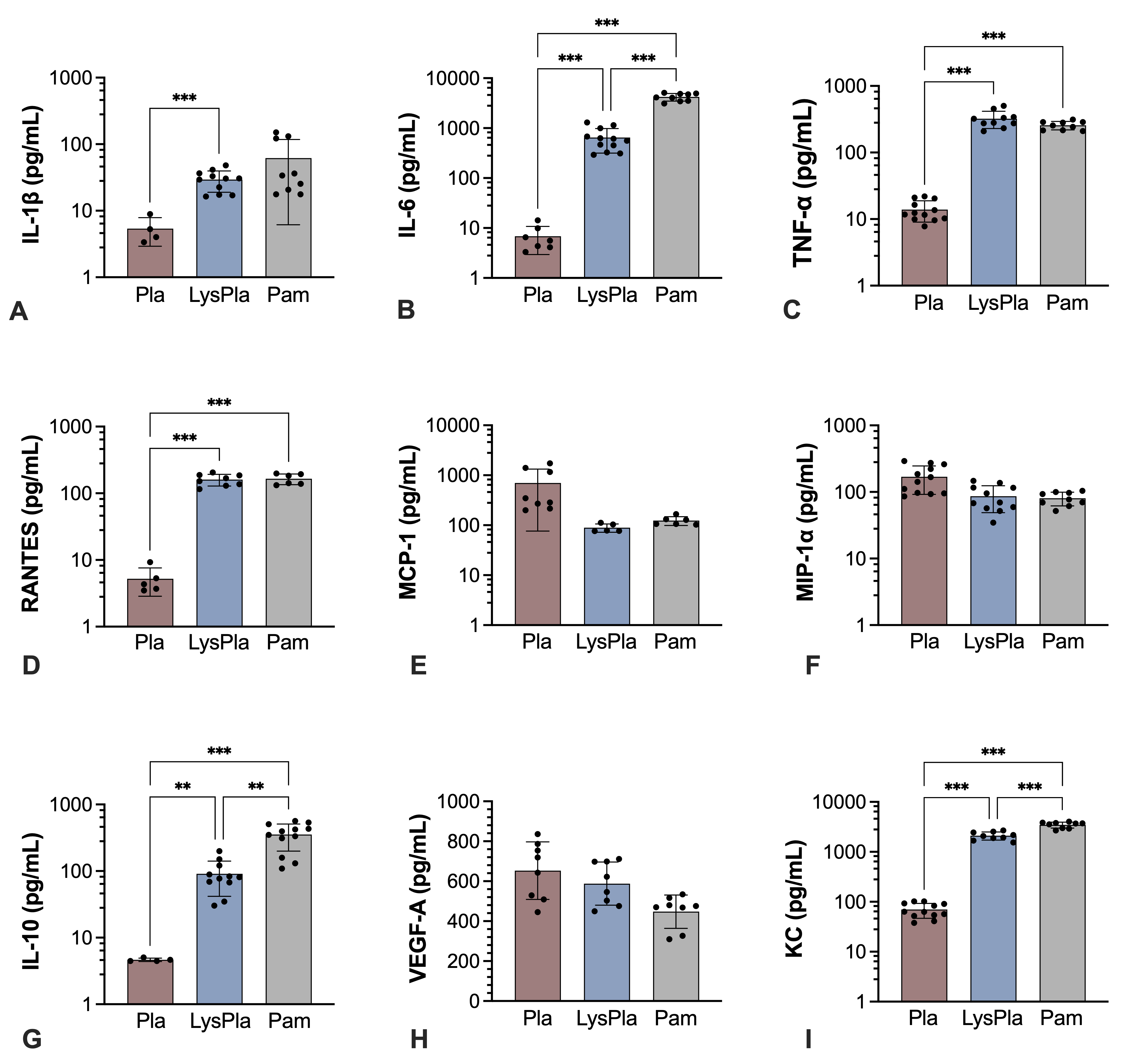


Supplementary Figure 4. Concentration of pro-inflammatory (A-F), anti-inflammatory (G) and angiogenic (H,I) cytokines in supernatant of WT BMDMs cultured for 24 hours on Teflon™ AF with adsorbed 10% plasma (Pla), 10% lysate in plasma (LysPla) or with Pam3CSK4 (Pam). Results are displayed as mean ± SD for four independent experiments, each containing 3 replicates (n = 3 - 12). A Brown-Forsythe and Welch ANOVA and Dunnett T3 post-hoc tests were used to determine significant differences among conditions, with α = 0.05. * p < 0.05, ** p < 0.01 and *** p < 0.001.

**Supplementary Table 1.** List of known protein DAMPs and their receptors (adapted from [1]), and the manual search terms used to identify DAMPs within the list of differentially expressed proteins in plasma vs lysate in plasma.

| **Protein DAMPs** | **Receptors** | **Search Term(s)** | **Identified in Adsorbed Proteins** |
| --- | --- | --- | --- |
| Actin | TREM1, DNGR1 | Actin | 3 |
| Annexin 1 | FPR1 | Annexin | 1 |
| Apolipoproteins (APOA1, APOA2, APOB, APOE, APOJ) | TREM2 | Apolipoprotein, APOA1, APOA2, APOB, APOE, APOJ | 4 |
| Amyloid β | TLR2, NLRP3, RAGE, FPR2 | Amyloid | 0 |
| Biglycan | TLR2, TLR4 | Biglycan | 1 |
| Calreticulin | CD91 | Calreticulin | 1 |
| Cardiolipin | TREM2 | Cardiolipin | 0 |
| Cathepsin G | FPR1 | Cathepsin, Cathepsin G | 0 |
| Cyclophilin A | CD147 | Cyclophilin | 0 |
| Decorin | TLR2, TLR4 | Decorin | 0 |
| Eosinophil-derived neurotoxin | TLR2 | Neurotoxin | 0 |
| Fibrinogen | TLR4 | Fibrinogen | 3 |
| Fibronectin | TLR2, TLR4 | Fibronectin | 1 |
| HDL | TREM2 | lipoprotein | 0 |
| Histones | TLR2, TLR4, TLR9 | histone | 16 |
| HMGB1 | TLR2, TLR4, RAGE, TIM3, TREM1 | HMGB1 | 1 |
| HMGN1 | TLR4 | HMGN1 | 0 |
| HSP | TLR2, TLR4, TREM1, CD91, TREM2 | Heat Shock, Hsp | 14 |
| IL-1α | IL-1R | Interleukin | 0 |
| IL-33 | ST2 | Interleukin | 0 |
| Lactoferrin | TLR4 | Lactoferrin | 0 |
| LDL | TREM2 | Lipoprotein | 0 |
| LL-37 | FPR2 | LL-37, hCAP-18 | 0 |
| Lipoprotein a | TREM2 | Lipoprotein | 0 |
| Neutrophil elastase | TLR4 | Elastase | 0 |
| ox-LDL | TLR4, FPR2 | LDL, lipoprotein |  |
| Peroxiredoxin 1 | TLR4 | Peroxiredoxin 1 | 1 |
| PGLYRP1 | TREM1 | Peptidoglycan Recognition Protein 1, |  |
| S100s | TLR2, TLR4, RAGE | S100 | 3 |
| SAA | TLR2, TLR4, FPR2 | SAA, serum amyloid | 1 |
| SAP130 | MINCLE | SAP130, Sin3A Associated Protein 130 | 0 |
| *Continued* |  |  |  |
| **Protein DAMPs** | **Receptors** | **Search Term(s)** | **Identified in Adsorbed Proteins** |
| Sphingomylin | TREM2 | Sphingomylin | 0 |
| Surfactant protein A/D | TLR2, TLR4 | Surfactant | 0 |
| TAFA4 | FPR1 | TAFA4 | 0 |
| Tenascin-C | TLR4 | Tenascin | 0 |
| TFAM | RAGE, TLR9 | TFAM | 0 |
| Versican | TLR2 | Versican | 0 |
| VLDL | TREM2 | Lipoprotein | 0 |
| β-defensins | TLR2, TLR4 | Defensin | 0 |
| β2-glycoprotein (beta2GPI) | TLR2 | Beta 2-glycoprotein, glycoprotein | 1 |

[1] T. Gong, L. Liu, W. Jiang, R. Zhou, DAMP-sensing receptors in sterile inflammation and inflammatory diseases, Nat Rev Immunol 20(2) (2020) 95-112.
